#  Novel Thiazolidinone-Azole Hybrids: Design, Synthesis and Antimycobacterial Activity Studies

**Published:** 2016

**Authors:** Barbaros Eroglu, Keriman Ozadali-Sari, Oya Unsal-Tan, Sriram Dharmarajan, Perumal Yogeeswari, Ayla Balkan

**Affiliations:** a *Department of Pharmaceutical Chemistry, Faculty of Pharmacy, Hacettepe University, Ankara, Turkey.*; b *Medicinal Chemistry & Antimycobacterial Research Laboratory, Pharmacy Group, Birla Institute of Technology & Science – Pilani, Hyderabad Campus, Jawahar Nagar, Hyderabad 500 078, Andhra Pradesh, India.*

**Keywords:** Thiazolidin-4-one, Triazole, Imidazole, Antimycobacterial activity

## Abstract

To develop novel antimycobacterial agents, a new series of thiazolidinone-azole hybrids 4a-b, 5a-b and 6-13 were designed and synthesized. Thiazolidin-4-ones (4a-b and 5a-b) were obtained by the reaction of *Schiff* bases and hydrazones (2a-b and 3a-b) with mercaptoacetic acid. 5-Benzylidene derivatives (6-13) were gained by treatment of 5a-b with appropriate benzaldehydes according to *Knoevenagel* condensation. To evaluate their structures ^1^H NMR, IR, mass spectrometry and elemental analysis data were used. The target compounds were screened for their antimycobacterial activity against M. tuberculosis H37Rv strain using the microplate alamar blue assay method. Among them, 6, 10 and 12 (MIC: 14.27-14.74 μM) were found as most active compounds in the series. It was seen that both phenylamino and benzylidene substitutions on thiazolidin-4-one ring caused an improvement in the antimycobacterial activity.

## Introduction

Tuberculosis (TB) is one of the most common infectious diseases known to man. According to the WHO report in 2012, there were almost 9 million new cases of tuberculosis and 1.4 million tuberculosis deaths (1). The problems with current TB treatment are complex and include: a prolonged standard course regimen of six months, which often result in patient noncompliance; emergency of extremely drug-resistant tuberculosis (XDR-TB) strains; lack of effective drugs against the latent state (2-4). Therefore, there is an urgent need for antitubercular agents with improved properties such as enhanced activity against MDR strains, reduced toxicity, shortened duration of treatment.

Thiazolidin-4-one is an important scaffold known to be associated with several biological activities. It is known that (-) 2-(5-carboxypentyl)thiazolidin-4-one (actithiazic acid) isolated from the culture broth of a strain of streptomyces has antimycobacterial activity (5). This discovery has prompted researchers to focus on new thiazolidin-4-one derivatives as potential antimycobacterial agents (6-10). On the other hand, it has been extensively reported that many structurally diverse compounds having azole moieties such as imidazole (11-14), triazole (15-17), thiazole (18, 19), oxazole (20), pyrazole (21, 22), isoxazole (23), oxadiazole (24) showed antimycobacterial activity. In recent years, one of the important strategies used in antitubercular drug development studies is hybridization of the biologically active structures. Considering this strategy, it was reported to achieve a highly active molecule by hybridization of thiazolidin-4-one ring with various active structures (25).

These facts have led us to study on thiazolidinone-azole hybrid compounds which are expected to show antitubercular activity.

## Experimental

*Chemistry*

Melting points were determined with a Thomas-Hoover Capillary Melting Point Apparatus (Philadelphia, PA, USA) and are uncorrected. ATR-FTIR spectra were obtained using the MIRacle ATR accessory (Pike technologies) in conjunction with a Spectrum BX FTIR spectrometer (Perkin Elmer) and were reported in cm^-1^. The ^1^H NMR (400 MHz) spectra (DMSO-d_6_) were recorded on a Varian Mercury 400 FT NMR spectrophotometer (Varian Inc, Palo Alto, CA, USA) using TMS as an internal reference (Chemical shift represented in δ ppm).The ESI-MS spectra were measured on a micromass ZQ-4000 single quadruple mass spectrometer. Elemental analyses (C, H, N) were performed on Leco CHNS 932 analyzer (USA). 

*General procedure for the preparation of Schiff bases 2a-b and hydrazones 3a-b*

Equimolar amounts of an appropriate benzaldehyde and anilin/phenylhydrazine were refluxed in methanol in presence of acetic acid (1 drop) as a catalytic reagent for 4 h. The solvent was evaporated and the crude mixture was used without any purification in the next step.

*General procedure for the preparation of 3-phenyl-2-[4-(1H-1,2,4-triazol/imidazol-1-yl)phenyl]-1,3-thiazolidin-4-ones 4a-b and 3-(phenylamino)-2-[4-(1H-1,2,4-triazol/imidazol-1-yl)phenyl]-1,3-thiazolidin-4-ones 5a-b*

A mixture of *Schiff* bases or hydrazones 2a-b or 3a-b (1 mmol) and excess of mercaptoacetic acid (1 mL) was heated at 60-80 ºC until the reaction was completed. Ethyl acetate (5 mL) was added, the organic layer was washed with saturated NaHCO_3_ (3x20 mL), water (1x10 mL), dried with Na_2_SO_4_ and concentrated to give an oily residue. The oily residue was purified by column chromatography on silica gel using hexane-ethyl acetate as eluent (3:7).

*3-phenyl-2-[4-(1H-1,2,4-triazol-1-yl)phenyl]-1,3-thiazolidin-4-one 4a*

53% yield, mp 176-8 ^ο^C. ^1^H NMR (DMSO-d_6_, 400 MHz); δ 9.25 (1H; s; triazole), 8.21 (1H; s; triazole), 7.77 (2H; d; Ar-H_3_‴ and H_5_‴ *J*: 8.4 Hz), 7.60 (2H; d; Ar-H_2_‴ and H_6_‴ *J*: 8.4 Hz), 7.36-7.28 (4H; m; Ar-H_2_*ꞌ*, H_3_*ꞌ*, H_5_*ꞌ* and H_6_*ꞌ*), 7.16-7.14 (1H; m; Ar-H_4_*ꞌ*), 6.61 (1H; s; thiazol., H_2_), 4.08 (1H; dd; thiazol., H_5a_, *J*: 1.6 Hz,* J*: 15.6 Hz), 3.92 (1H; d; thiazol., H_5b_, *J*: 15.6 Hz). IR; cm^-1^ 1674 (C=O). ESI-MS (m/z); 345 [M+Na]^+^, 323 [M+H]^+^. Anal. Calcd. for C_17_H_14_N_4_OS: C, 63.34; H, 4.38; N, 17.38; S, 9.94. Found: C, 62.28; H, 4.45; N, 17.60; S, 9.62.

*3-phenyl-2-[4-(1H-imidazol-1-yl)phenyl]-1,3-thiazolidin-4-one 4b*

47% yield, mp 75-77 ^ο^C. ^1^H NMR (DMSO-d_6_, 400 MHz); δ 8.22 (1H; s; imidazole), 7.71 (1H; s; imidazole), 7.57-7.52 (4H; m; Ar-H_2_‴, H_3_‴, H_5_‴ and H_6_‴), 7.36-7.28 (4H; m; Ar-H_2_*ꞌ*, H_3_*ꞌ*, H_5_*ꞌ* and H_6_*ꞌ*), 7.17-7.13 (1H; m; Ar-H_4_*ꞌ*), 7.07 (1H; s; imidazole), 6.60 (1H; s; thiazol., H_2_), 4.07 (1H; dd; thiazol., H_5a_, *J*: 1.2 Hz*, J*: 15.6 Hz), 3.90 (1H; d; thiazol., H_5b_, *J*: 15.6 Hz). IR; cm^-1^ 1681 (C=O). ESI-MS (m/z); 344 [M+Na]^+^, 322 [M+H]^+^. Anal. Calcd. for C_18_H_15_N_3_OS: C, 67.27; H, 4.70; N, 13.07; S, 9.98. Found: C, 62.48; H, 4.57; N, 13.43; S, 10.08.

*3-(phenylamino)-2-[4-(1H-1,2,4-triazol-1-yl)phenyl]-1,3-thiazolidin-4-one 5a*

48% yield, mp 228-30 ^ο^C (dec.).^1^H NMR (DMSO-d_6_, 400 MHz); δ 9.32 (1H; s; triazole), 8.25 (1H; s; triazole), 8.22 (1H; s; NH), 7.88 (2H; d; Ar-H_3_‴ and H_5_‴ *J*: 8.4 Hz), 7.60 (2H; d; Ar-H_2_‴ and H_6_‴, *J*: 8.4 Hz), 7.16 (2H; t; Ar-H_3_*ꞌ *and H_5_*ꞌ*), 6.75 (1H; t; Ar-H_4_*ꞌ*), 6.67 (2H; d; Ar-H_2_*ꞌ* and H_6_*ꞌ*, *J*: 7.6 Hz), 5.95 (1H; d; thiazol., H_2_, *J*: 1.2 Hz), 3.98 (1H; dd; thiazol., H_5a_, *J*: 1.6 Hz, *J*: 15.6 Hz), 3.79 (1H; d; thiazol., H_5b_, *J*: 15.6 Hz). IR; cm^-1 ^3220 (NH), 1696 (C=O). ESI-MS (m/z); 360 [M+Na]^+^, 338 [M+H]^+^. Anal. Calcd. for C_17_H_15_N_5_OS: C, 60.52; H, 4.48; N, 20.76; S, 9.50. Found: C, 60.31; H, 4.61; N, 20.54; S, 9.65.

*3-(phenylamino)-2-[4-(1H-imidazol-1-yl)phenyl]-1,3-thiazolidin-4-one 5b*

45% yield, mp 163-4 ^ο^C.^1^H NMR (DMSO-d_6_, 400 MHz); δ 8.28 (1H; s; imidazole), 8.22 (1H; s; NH), 7.77 (1H; s; imidazole), 7.66 (2H; d; Ar-H_3_‴ and H_5_‴ *J*: 8.8 Hz), 7.54 (2H; d; Ar-H_2_‴and H_6_‴, *J*: 8.8 Hz), 7.16 (2H; t; Ar-H_3_*ꞌ* and H_5_*ꞌ*), 7.11 (1H; s; imidazole), 6.75 (1H; t; Ar-H_4_*ꞌ*), 6.67 (2H; d; Ar-H_2_*ꞌ* and H_6_*ꞌ*, *J*: 8.8 Hz), 5.94 (1H; d; thiazol., H_2_, *J*: 1.6 Hz), 3.97 (1H; dd; thiazol., H_5a_, *J*: 1.6 Hz, *J*: 15.6 Hz), 3.79 (1H; d; thiazol., H_5b_, *J*:15.6 Hz). IR; cm^-1^ 3170 (NH), 1681 (C=O). ESI-MS (m/z); 359 [M+Na]^+^, 337 [M+H]^+^. Anal. Calcd. for C_18_H_16_N_4_OS: C, 64.27; H, 4.79; N, 16.65; S, 9.53. Found: C, 64.47; H, 4.58; N, 16.83; S, 9.76.

*General procedure for the preparation of 5-benzylidene/substitutedbenzylidene-3-(phenylamino)-2-[4-(1H-1,2,4-triazol/imidazol-1-yl)phenyl]-1,3-thiazolidin-4-ones 6-13*

A mixture of 3-(phenylamino)-2-[4-(1*H*-1,2,4-triazol/imidazol-1-yl)phenyl]-1,3-thiazolidin-4-ones 5a-b (10 mmol) and 4-nonsubstituted/substitutedbenzaldehyde (10 mmol) was refluxed in 30 mL of ethanol in the presence of potassium hydroxide (15 mmol) for 8 h. The solvent was evaporated under reduced pressure and obtained oily residue was crystallized from appropriate solvent.

*5-Benzylidene-3-(phenylamino)-2-[4-(1H-1,2,4-triazol-1-yl)phenyl]-1,3-thiazolidin*

*-4-one 6*

38% yield, mp 156-7 ^ο^C.^1^H NMR (DMSO-d_6_, 400 MHz); δ 9.29 (1H; s; triazole), 8.46 (1H; s; NH), 8.22 (1H; s; triazole), 7.88 ( 2H; d; Ar-H_2_″and H_6_″ *J*: 8.4 Hz), 7.59 (2H; d; Ar-H_3_‴ and H_5_‴ *J*: 8.4 Hz), 7.56 (2H; d; Ar-H_3_″ and H_5_″*J*: 7.2 Hz), 7.51 (1H; s; CH=), 7.46 (2H; d; Ar-H_2_‴and H_6_‴ *J*: 8.4 Hz), 7.35 (1H; t; Ar-H_4_″), 7.15 (2H; t; Ar-H_3_*ꞌ* and H_5_*ꞌ*), 6.75 (1H; t; Ar-H_4_*ꞌ*), 6.65 (2H; d; Ar-H_2_*ꞌ* and H_6_*ꞌ* *J*: 8.0 Hz), 6.33 (1H; s; CH). IR; cm^-1^ 3266 (NH), 1676 (C=O). ESI-MS (m/z); 448 [M+Na]^+^, 426 [M+H]^+^. Anal. Calcd. for C_24_H_19_N_5_OS: C, 67.74; H, 4.50; N, 16.46; S, 7.53. Found: C, 67.37; H, 4.54; N, 16.05; S, 6.81.

*5-(4-Bromobenzylidene)-3-(phenylamino)-2-[4-(1H-1,2,4-triazol-1-yl)phenyl]-1,3-thiazolidin-4-one 7*

57% yield, mp 192-3 ^ο^C.^1^H NMR (DMSO-d_6_, 400 MHz); δ 9.30 (1H; s; triazole), 8.50 (1H; s; NH), 8.23 (1H; s; triazole), 7.88 ( 2H; d; Ar-H_2_″ and H_6_″, *J*: 8.4 Hz), 7.65 (2H; d; Ar-H_3_‴ and H_5_‴ , *J*: 8.4 Hz), 7.59 (2H; d; Ar-H_3_″ and H_5_″, *J*: 8.4 Hz), 7.51 (2H; d; Ar-H_2_‴ and H_6_‴ , *J*: 8.4 Hz), 7.48 (1H; s; CH=), 7.15 (2H; t; Ar-H_3_*ꞌ*and H_5_*ꞌ*), 6.75 (1H; t; Ar-H_4_*ꞌ*), 6.44 (2H; d; Ar-H_2_*ꞌ* and H_6_*ꞌ*, *J*: 7.6 Hz), 6.35 (1H; s; CH). IR; cm^-1^ 3266 (NH), 1681 (C=O). ESI-MS (m/z); 544 [M+K+2]^+^, 528 [M+Na+2]^+^. Anal. Calcd. for C_24_H_18_BrN_5_OS.1 H_2_O: C, 55.18; H, 3.86; N, 13.41; S, 6.14. Found: C, 54.84; H, 3.73; N, 13.41; S, 6.64.

*5-(4-Methylbenzylidene)-3-(phenylamino)-2-[4-(1H-1,2,4-triazol-1-yl)phenyl]-1,3-thiazolidin-4-one 8*

29% yield, mp 161-2 ^ο^C.^1^H NMR (DMSO-d_6_, 400 MHz); δ 9.31 (1H; s; triazole), 8.48 (1H; s; NH), 8.24 (1H; s; triazole), 7.89 ( 2H; d; Ar-H_2_″ and H_6_″ , *J*: 7.2 Hz), 7.59 (2H; d; Ar-H_3_‴ and H_5_‴ , *J*: 7.6 Hz), 7.48 (1H; s; CH=), 7.46 (2H; d; Ar-H_3_″ and H_5_″ , *J*: 8.4 Hz), 7.28 (2H; d; Ar-H_2_‴ and H_6_‴ , *J*: 8.4 Hz), 7.15 (2H; t; Ar-H_3_*ꞌ* and H_5_*ꞌ*), 6.76 (1H; t; H_4_*ꞌ*), 6.66 (2H; d; Ar-H_2_*ꞌ* and H_6_*ꞌ*, *J*: 8.0 Hz), 6.33 (1H; s; CH), 3.34 (3H; s; CH_3_). IR; cm^-1^ 3175 (NH), 1683 (C=O). ESI-MS (m/z); 478 [M+K]^+^, 462 [M+Na]^+^, 440 [M+H]^+^. Anal. Calcd. for C_25_H_21_N_5_OS.1/2 H_2_O: C, 66.94; H, 4.94; N, 15.61; S, 7.15. Found: C, 67.13; H, 4.64; N, 15.69; S, 7.28.

*5-(4-Chlorobenzylidene)-3-(phenylamino)-2-[4-(1H-1,2,4-triazol-1-yl)phenyl]-1,3-thiazolidin-4-one 9*

43% yield, mp 163-4 ^ο^C.^1^H NMR (DMSO-d_6_, 400 MHz); δ 9.29 (1H; s; triazole), 8.47 (1H; s; NH), 8.22 (1H; s; triazole), 7.88 (2H; d; Ar-H_2_″ and H_6_″ , *J*: 8.4 Hz), 7.65 (2H; d; Ar-H_3_‴ and H_5_‴, *J*: 8.4 Hz), 7.59 (2H; d; Ar-H_3_″ and H_5_″ , *J*: 8.4 Hz), 7.51 (2H; d; Ar-H_2_‴ and H_6_‴, *J*: 8.4 Hz), 7.48 (1H; s; CH=), 7.15 (2H; t; Ar-H_3_*ꞌ *and H_5_*ꞌ*), 6.75 (1H; t; Ar-H_4_*ꞌ*), 6.65 (2H; d; Ar-H_2_*ꞌ* and H_6_*ꞌ*, *J*: 7.2 Hz), 6.35 (1H; s; CH). IR; cm^-1^ 3268 (NH), 1676 (C=O). ESI-MS (m/z); 500 [M+K+2]^+^, 482 [M+Na+2]^+^. Anal. Calcd. for C_24_H_18_ClN_5_OS.1/2 H_2_O: C, 61.47; H, 4.08; N, 14.93; S, 6.84. Found: C, 61.94; H, 3.99; N, 15.22; S, 6.82.

*5-Benzylidene-3-(phenylamino)-2-[4-(1H-imidazol-1-y)phenyl]-1,3-thiazolidin-4-one 10*

41% yield, mp 189-90 ^ο^C.^1^H NMR (DMSO-d_6_, 400 MHz); δ 8.48 (1H; s; NH), 8.26 (1H; s; imidazole), 7.74 (1H; s; imidazole), 7.68 ( 2H; d; Ar-H_2_″ and H_6_″, *J*: 8.4 Hz), 7.57-7.45 (7H; m; CH=, Ar-H_2_‴, H_3_‴, H_5_‴, H_6_‴, H_3_″ and H_5_″), 7.36 (1H; t; Ar-H_4_″), 7.18 (2H; t; Ar-H_3_*ꞌ* and H_5_*ꞌ* , 7.09 (1H; s; imidazole), 6.76 (1H; t; Ar-H_4_*ꞌ* ), 6.66 (2H; d; Ar-H_2_*ꞌ* and H_6_*ꞌ* , *J*: 8.0 Hz), 6.33 (1H; s; CH). IR; cm^-1^ 3288 (NH), 1698 (C=O). ESI-MS (m/z); 447 [M+Na]^+^, 425 [M+H]^+^. Anal. Calcd. for C_25_H_20_N_4_OS: C, 70.73; H, 4.75; N, 13.20; S, 7.55. Found: C, 70.67; H, 4.99; N, 13.72; S, 7.43.

*5-(4-Bromobenzylidene)-3-(phenylamino)-2-[4-(1H-imidazol-1-y)phenyl]-1,3-thiazolidin-4-one 11*

61% yield, mp 223-4 ^ο^C.^1^H NMR (DMSO-d_6_, 400 MHz); δ 8.62 (1H; s; NH), 8.53 (1H; s; imidazole), 7.89 (1H; s; imidazole), 7.74 ( 2H; d; Ar-H_2_″ and H_6_″, *J*: 8.4 Hz), 7.68 (2H; d; Ar-H_3_‴ and H_5_‴, *J*: 8.4 Hz), 7.59 (2H; d; Ar-H_3_″ and H_5_″, *J*: 8.8 Hz), 7.53 (2H; d; Ar-H_2_‴ and H_6_‴, *J*: 9.2 Hz), 7.51 (1H; s; CH=), 7.31 (1H; s; imidazole), 7.18 (2H; t; Ar-H_3_*ꞌ* and H_5_*ꞌ*), 6.78 (1H; t; Ar-H_4_*ꞌ*), 6.68 (2H; d; Ar-H_2_*ꞌ* and H_6_*ꞌ*, *J*: 8.0 Hz), 6.38 (1H; s; CH). IR; cm^-1^ 3266 (NH), 1696 (C=O). ESI-MS (m/z); 527 [M+Na+2]^+^, 505 [M+H+2]^+^. Anal. Calcd. for C_25_H_19_BrN_4_OS.3 H_2_O: C, 53.86; H, 4.52; N, 10.05; S, 5.75. Found: C, 54.00; H, 4.12; N, 10.43; S, 5.85.

*5-(4-Methylbenzylidene)-3-(phenylamino)-2-[4-(1H-imidazol-1-y)phenyl]-1,3-thiazolidin-4-one 12*

47% yield, mp 215-6 ^ο^C.^1^H NMR (DMSO-d_6_, 400 MHz); δ 8.47 (1H; s; NH), 8.23 

(1H; s; imidazole), 7.75 (1H; s; imidazole), 7.70 ( 2H; d; Ar-H_2_″ and H_6_″, *J*: 8.4 Hz), 

7.55 (2H; d; Ar-H_3_‴ and H_5_‴, *J*: 8.4 Hz), 7.49 (1H; s; CH=), 7.47 (2H; d; Ar-H_3_″ and H_5_″, 

*J*: 8.0 Hz), 7.29 (2H; d; Ar-H_2_‴ and H_6_‴, *J*: 8.4 Hz), 7.18 (2H; t; Ar-H_3_*ꞌ* and H_5_*ꞌ*), 7.11 (1H; s; imidazole), 6.77 (1H; t; Ar-H_4_*ꞌ*),6.68 (2H; d; Ar-H_2_*ꞌ* and H_6_*ꞌ*, *J*: 8.0 Hz), 6.32 (1H; s; CH), 3.34 (3H; s; CH_3_). IR; cm^-1^ 3288 (NH), 1695 (C=O). ESI-MS (m/z); 477 [M+K]^+^, 439 [M+H]^+^. Anal. Calcd. for C_26_H_22_N_4_OS.2 H_2_O: C, 65.80; H, 5.52; N, 11.81; S, 6.76. Found: C, 65.25; H, 5.21; N, 11.73; S, 6.74.

*5-(4-Chlorobenzylidene)-3-(phenylamino)-2-[4-(1H-imidazol-1-y)phenyl]-1,3-thiazolidin-4-one 13*

53% yield, mp 217-8 ^ο^C.^1^H NMR (DMSO-d_6_, 400 MHz); δ 8.51 (1H; s; NH), 8.28 (1H; s; imidazole), 7.77 (1H; s; imidazole), 7.74 ( 2H; d; Ar-H_2_″ and H_6_″, *J*: 8.4 Hz), 7.68 (2H; d; Ar-H_3_‴ and H_5_‴ , *J*: 8.4 Hz), 7.59 (2H; d; Ar-H_3_″ and H_5_″, *J*: 8.8 Hz), 7.53 (2H; d; Ar-H_2_‴ and H_6_‴ , *J*: 9.2 Hz), 7.51 (1H; s; CH=), 7.18 (2H; t; Ar-H_3_*ꞌ* and H_5_*ꞌ* ), 7.11 (1H; s; imidazole), 6.78 (1H; t; Ar-H_4_*ꞌ* ), 6.68 (2H; d; Ar-H_2_*ꞌ* and H_6_*ꞌ* , *J*: 8.0 Hz), 6.36 (1H; s; CH). IR; cm^-1^ 3268 (NH), 1697 (C=O). ESI-MS (m/z); 483 [M+Na+2]^+^, 461 [M+H+2]^+^. Anal. Calcd. for C_25_H_19_ClN_4_OS.1/2 H_2_O: C, 64.16; H, 4.31; N, 11.97; S, 6.85. Found: C, 64.27; H, 5.02; N, 11.21; S, 6.51.

*Antimycobacterial Activity Assay*

The target compounds were tested for their antimycobacterial activity in vitro against M. tuberculosis H_37_R_v_ using the microplate alamar blue assay (MABA) method (27) in duplicate. Isoniazid, rifampin, ethambutol and ciprofloxacin were used as positive and DMSO as negative control. Compound stock solutions were prepared in DMSO. Sterile deionized water (200 μL) was added to all outer-perimeter wells of sterile 96-well plates to minimize evaporation of the medium in the test wells during incubation. The wells received 100 μL of Middlebrook 7H9GC broth and two fold serial dilutions of the target compounds/positive controls were prepared in a volume of 100 μL directly on the plate. 100 μL of *MTB* inoculum was added to the wells. The plates were sealed with parafilm and were incubated at 37 °C for five days. 50 μL of a freshly prepared 1:1 mixture of Alamar Blue (Accumed International, Westlake, Ohio) reagent and 10% Tween 80 was added to the plates and incubated at 37 °C for 24 h. A blue colour in the well was interpreted as no growth, and a pink colour was scored as growth. The MIC was determined as the lowest drug concentration which prevented a colour change from blue to pink. MICs of the compounds were reported in Table 1. 

*Conflict of Interest*

We declare that we have no conflict of interest with respect to this study.

## Results and Discussion

*Chemistry*

**Figure 1:**
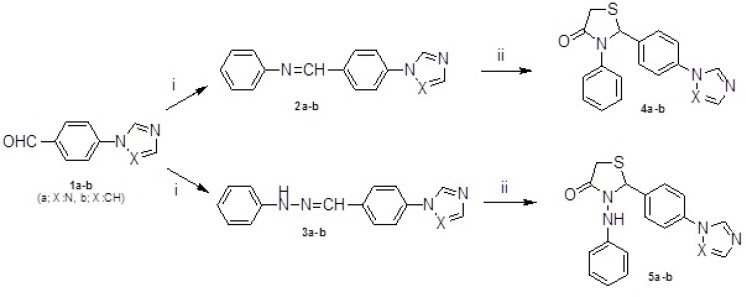
Synthesis of 4a-b and 5a-b. Reagents and conditions: (i) aniline/phenylhydrazine, CH_3_COOH, methanol, reflux; (ii) mercaptoacetic acid, 60 ºC

**Figure 2:**
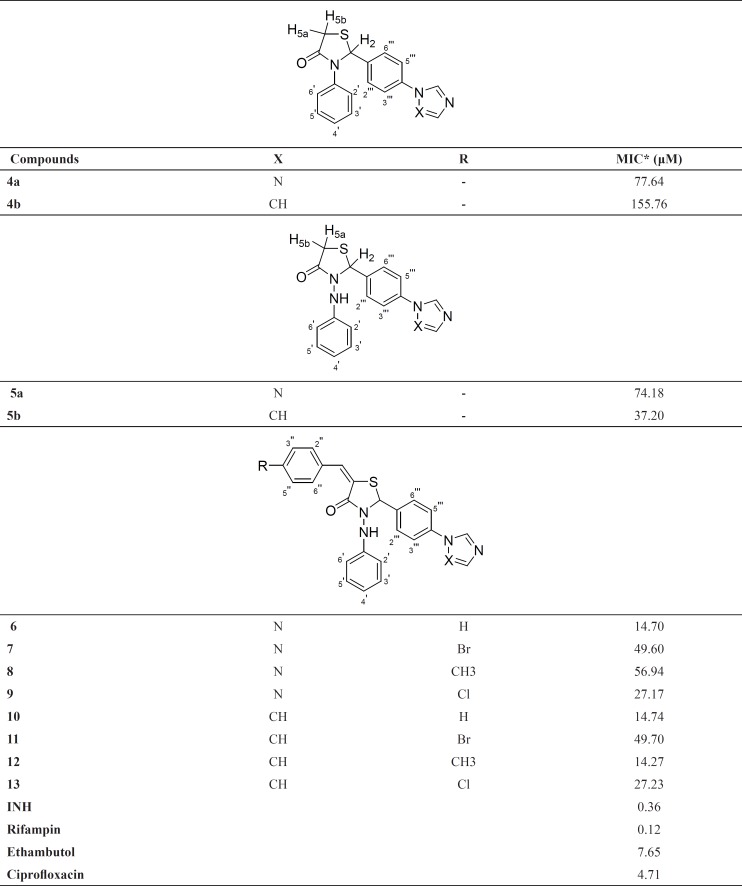
Synthesis of 6-13. Reagent and condition: (i) appropriate benzaldehyde, KOH, ethanol, reflux

The starting compounds, 4-(1*H*-1,2,4-triazol/imidazol-1-yl)benzaldehydes 1a-b were synthesized in accordance with the method described in the literature (26). The intermediate compounds, *Schiff* bases and hydrazones (2a-b and 3a-b), were obtained by condensation of 1a-b with anilin or phenylhydrazine respectively. Reaction of 2a-b and 3a-b with mercaptoacetic acid yielded the thiazolidin-4-ones 4a-b and 5a-b (Figure 1). Compounds 6-13 were gained by treatment of 5a-b with appropriate benzaldehydes according to *Knoevenagel* condensation as outlined in Figure 2. The structures of the target compounds (4a-b, 5a-b and 6-13) were confirmed by IR, ^1^H NMR, mass spectrometry and elemental analysis.

**Table 1:** Antimycobacterial activities of 4a-b, 5a-b and 6-13

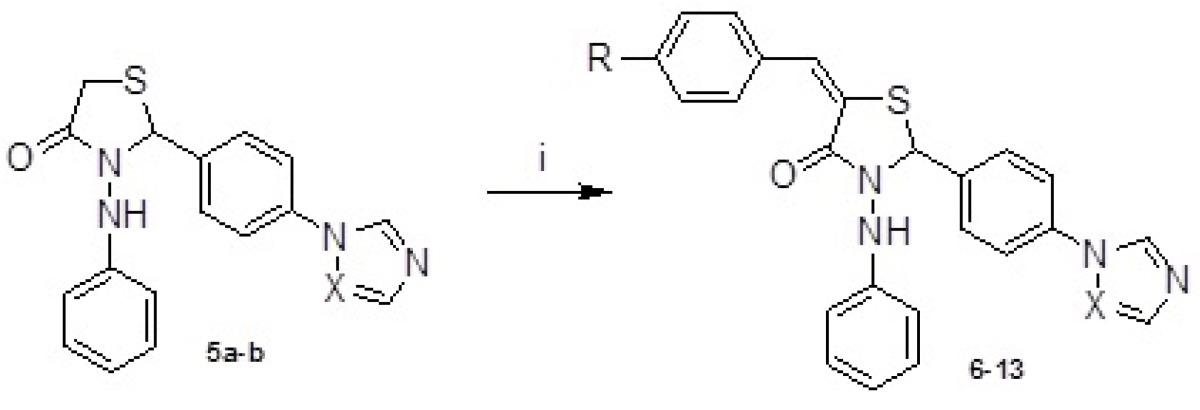

In the IR spectra of compound 4a-b and 5a-b, the presence of the C=O stretching bands at around 1680 cm^-1^ indicated the thiazolidin-4-one ring closure. In the ^1^H NMR spectra of these compounds, the signals of nonequivalence methylenic protons of the thiazolidin-4-one ring were observed as two doublets at around 4.0 ppm (H_A_) and as doublet at around 3.9 ppm (H_B_) because of geminal (*J*_AB_ of 15.6 Hz) and long range coupling (~*J*_Ax_ of 1.6 Hz) with C_2_ proton. Furthermore, the signal of the C_2_ proton of the thiazolidin-4-one ring was seen as singlet or doublet at around 6.0 ppm. In the ^1^H NMR spectra of 6-13, disappearance of the signals of methylenic protons and formation of a new signal at around 7.5 ppm proved benzylidene substitution to the thiazolidin-4-one ring. In the mass spectra, all compounds displayed molecular ion peaks which confirmed their molecular weight.

*Antimycobacterial Activity*

The target compounds (4a-b, 5a-b and 6-13) were evaluated for their antimycobacterial activity *in-vitro* against Mycobacterium tuberculosis H_37_R_v_ using the microplate alamar blue assay method (27) in duplicate. The results of the antimycobacterial activity (MIC values) were reported in Table 1.

When Table 1. were examined, it was seen that introduction of phenylamino group (5a with MIC of 74.18 μM and 5b with MIC of 37.20 μM) instead of phenyl group (4a with MIC of 77.64 μM and 4b with MIC of 155.76 μM) to 3th position of thiazolidin-4-one ring contributed to the activity, although this contribution was very limited for triazole substituted derivatives.

These results led us to focus on 3-(phenylamino)-1,3-thiazolidin-4-ones. As a continuation of our study, we condensed several benzaldehydes to obtain 5-(substitutedbenzylidene)-3-(phenylamino)-1,3-thiazolidin-4-ones (6-13). When we compared the activity results of 5a and 5b with 6-9 and 10-13 respectively, it was observed that benzylidene substitution to the 3-(phenylamino)-1,3-thiazolidin-4-ones enhanced the activity (except 11). However 12 (MIC: 14.27 μM) was the most active derivative in this series, introducing the substituents at the 4^th^ position of the benzylidene ring decreased the activity in generally. According to the activity results of 6 and 10 (with MIC of 14.70 and 14.74 μM respectively), it can be assumed that non-substitutedbenzylidene structure is a favorable moiety for the activity. Comparing the compounds bearing triazole moiety (6-9) with that of imidazole analogues (10-13), it was seen that the type of the azole ring did not cause any remarkable difference on the activity except methyl substituted derivatives (8 with MIC of 56.94 μM and 12 with MIC of 14.74 μM). Furthermore, none of the compounds in the series were found as active as standard compounds (Table 1).

In summary, a series of novel thiazolidin-4-one derivatives 4a-b, 5a-b and 6-13 were designed, synthesized to evaluate their antimycobacterial activity. Among the target compounds, 6, 10 and 12 were found as most active compounds in the series. It was seen that both phenylamino and benzylidene substitutions on thiazolidin-4-one ring caused an improvement in the antimycobacterial activity. 
